# Supplementary material for: Content-rich biological network constructed by mining PubMed abstracts
Source: BMC Bioinformatics. 2004 Oct 8;5:147. doi: 10.1186/1471-2105-5-147 (PMC528731; doi:10.1186/1471-2105-5-147)
Supplement: Additional File 2 — The original results of the above study (non-essential files are deleted to keep the file size under the limit set by BMC bioinformatics). [file 1471-2105-5-147-S2.bz2 › chilibotAdditionalFile2/dip20/165ID8599116E572/html/AKAP5_PRKCA.html]

 


 **AKAP5** and **PRKCA** 
  
Found 15 abstracts in PubMed, retrieved 15.  
 

 What does Google say? 
 PDF only 
| .edu only 

---

**Interactive relationship** (e.g. stimulation, inhibition, etc)

**Non-interactive relationship** (e.g. studied together, co-existance, homology, etc.)

- A kinase  [ **AKAP5** ]  defective mutant of PKC alpha  [ **PRKCA** ]  down regulates in response to phorbol esters as effectively as the wild type protein when introduced into COS 1 cells.  Ref: 2060619 FEBS Lett, 1991
- A kinase  [ **AKAP5** ]  defective protein kinase C alpha  [ **PRKCA** ]  mutant is shown to be a phosphoprotein when expressed in COS 1 cells, indicating that intramolecular phosphorylation does not fully account for the phosphate content of protein kinase C alpha  [ **PRKCA** ] .  Ref: 1575696 Biochem J, 1992
- Moreover, a kinase  [ **AKAP5** ]  dead ATP binding mutant of PKC alpha  [ **PRKCA** ]  could not be depleted by TPA treatment.  Ref: 9447980 Mol Cell Biol, 1998
- Down regulation of a kinase  [ **AKAP5** ]  defective PKC alpha  [ **PRKCA** ] .  Ref: 2060619 FEBS Lett, 1991
